# Fenton vs. Intergrowth-21st: Postnatal Growth Assessment and Prediction of Neurodevelopment in Preterm Infants

**DOI:** 10.3390/nu13082841

**Published:** 2021-08-18

**Authors:** Miheret Yitayew, Nayef Chahin, Salem Rustom, Leroy R. Thacker, Karen D. Hendricks-Muñoz

**Affiliations:** 1 Department of Pediatrics, Children’s Hospital of Richmond, Virginia Commonwealth University, Richmond, VA 23298, USA; nayef.chahin@vcuhealth.org (N.C.); karen.hendricks-munoz@vcuhealth.org (K.D.H.-M.); 2 Division of Neonatal Medicine, Department of Pediatrics, School of Medicine, Virginia Commonwealth University, Richmond, VA 23298, USA; 3 Department of Biostatistics, Virginia Commonwealth University, Richmond, VA 23298, USA; rustoms@vcu.edu (S.R.); leroy.thacker@vcuhealth.org (L.R.T.)

**Keywords:** preterm infant, growth curve, intergrowth, Fenton, growth failure, neurodevelopment

## Abstract

Although the survival rate of preterm infants has improved over the years, growth failure and associated impaired neurodevelopmental outcome remains a significant morbidity. Optimal nutrition plays an important role in achieving adequate postnatal growth. Accurate growth monitoring of preterm infants is critical in guiding nutritional protocols. Currently, there is no consensus regarding which growth assessment tool is suitable for monitoring postnatal growth of preterm infants to foster optimal neurodevelopmental outcomes while avoiding future consequences of aggressive nutritional approaches including increased risk for cardiovascular disease and metabolic syndrome. A retrospective single center cohort study was conducted to compare the performance of two growth-assessment tools, Fenton and Intergrowth-21st (IG-21st) in the classification of size at birth, identification of impaired growth and predicting neurodevelopment. A total of 340 infants with mean gestational age of 30 weeks were included. Proportion of agreement between the two tools for identification of small for gestational age (SGA) was high 0.94 (0.87, 0.1) however, agreement for classification of postnatal growth failure at discharge was moderate 0.6 (0.52, 0.69). Growth failure at discharge was less prevalent using IG-21st. There was significant association between weight-based growth failure and poor neurodevelopmental outcomes at 12 and 24 months of age.

## 1. Introduction

Although the survival rate of the greater than 15 million preterm infants born annually has improved, they have higher morbidity with 20–45% having suboptimal neurodevelopment compared to infants born at term, further challenging clinicians in optimizing care to improve long-term outcomes [1,2,3,4]. Nutrition and growth are critical to brain development [5,6] and several studies have linked poor postnatal growth in infants to poor neurodevelopmental outcome [3,4,5,6]. For the preterm infant, growth trajectory is complicated with abrupt loss of maternal nutrients and later adaptation and dependence on postnatal intestinal absorption characterizing the complex biology of gastrointestinal development and varying feeding standards [7]. Clinical nutritional guidelines for the premature infant continue to be based on expert opinions, highlighting the lack of high-quality evidence in guiding clinicians to determine the exact type and timing of nutritional requirement of the preterm infant. According to the American Academy of Pediatrics’ guidance, adequate nutrition for the preterm infant is ideally designed to provide nutrients to approximate the rate of growth and composition of weight gain for a normal fetus of the same post-menstrual age [8]. However, this goal has proved challenging as extrauterine growth restriction remains a universal problem. This approach has been questioned repeatedly in recent years arguing that preterm infants who are in different physiologic, nutritional, and environmental conditions should not be compared to fetuses at the same gestational age [9]. Furthermore, it leads not only to the misclassification of most infants with growth failure, but also to aggressive nutritional approaches that have been linked to disproportional growth, aberrant adiposity, and increased risk for metabolic syndrome [10,11,12]. Despite creation of several growth charts and their subsequent modification in the past, monitoring growth and defining growth deficiency in preterm infants has been a constant challenge for neonatologists [9,13]. The adequacy of growth in neonatal intensive care unit (NICU) is currently assessed by using serial measures of weight, length, and head circumference plotted overtime on population growth charts and comparing growth trajectories with reference data. Current growth assessment charts that provide these reference data are based on fetal growth-based ross-sectional data lacking the ability to depict growth of preterm infants under optimal conditions. The Fenton growth chart is one of the commonly used reference charts based on size at birth, developed from several cross-sectional population studies data [14]. As it is not based on the longitudinal study, the change in weight after birth in preterm population was not included in the data of the Fenton charts and hence postnatal growth is compared to intrauterine growth for same post-menstrual age. In 2014 the International Fetal and Newborn Growth Consortium for the 21st Century (IG-21st) study used a prescriptive approach to describe normal fetal growth, preterm growth, and newborn nutritional status from eight geographically diverse population [15,16]. Although this growth-monitoring tool is based on a healthy preterm cohort and aims to provide a realistic and more appropriate international standard for monitoring of preterm infant’s growth, its universal adaption has been limited by lack of studies that evaluate its performance and functional impact. In this study we aim to compare the performance of IG-21st versus Fenton growth reference in identifying small for gestational Age (SGA) preterm infants, growth failure at discharge and its ability to predict impaired neurodevelopmental outcomes at 12 and 24 months of age in preterm infants.

## 2. Materials and Methods

This is a retrospective cohort study of preterm infants 24^0/7^ to 32^6/7^ weeks of gestation admitted to the neonatal intensive care unit at the Children’s Hospital of Richmond (CHoR)who survived to discharge between the years of 2014 and 2018. Infants with major congenital anomalies and chromosomal abnormalities including all aneuploidies, congenital brain malformations, and congenital defects that are require prolonged parenteral nutrition and are associated with poor growth were excluded. Data on infant demographics, anthropometry measurements, short-term neonatal outcomes and neurodevelopment assessment scores were obtained from medical records. Short-term neonatal outcomes included bronchopulmonary dysplasia (supplemental oxygen administration at 36 weeks of post-menstrual age), necrotizing enterocolitis (Bell stage ≥ II), sepsis (positive blood culture), retinopathy of prematurity (retinopathy requiring treatment), any intraventricular hemorrhage and periventricular leukomalacia. Growth parameters such as weight, length, and head circumference were measured at regular intervals by NICU nurses per unit protocol. Weight, length, and head circumference centiles and Z-scores were determined using bulk calculators for Fenton (2013) and IG-21st growth chart, which are publicly available at Fenton Growth Chart|University of Calgary (ucalgary.ca (accessed on 9 April 2021)), and INTERGROWTH-21st (ox.ac.uk (accessed on 9 April 2021)) respectively. While using IG-21st, we utilized newborn size for preterm infants’ chart to assess size at birth and the postnatal growth of preterm infants’ charts to assess postnatal growth. Neurodevelopment was assessed using Bayley Scales of Infant Development 3rd edition (BSID-III) at 12 and 24 months by trained and certified examiners. The primary outcome of the study was poor neurodevelopmental outcome defined as composite score < 85 in any of the development subsets tested either at 12 or 24 months. The study protocol was approved by the Virginia Commonwealth University Institutional Review Board.

### Statistical Analyses

Descriptive statistics is provided for demographic data and baseline characteristics. Median and IQR were used to summarize all continuous variables, while frequencies and percentages summarized all categorical variables. In addition, the preterm infant’s growth z-score in weight, length, and head circumference at birth was summarized by both Fenton and IG-21st. The Z-score at discharge, and the change in z-score between birth and discharge for all growth parameters was also summarized by each chart. Cohen’s Kappa (with 95% confidence intervals) was used to assess the agreement between Fenton and IG-21st, and paired *t*-tests were used to compare Z-scores between the two charts. Unadjusted Logistic regression models were constructed to quantify the differences in poor neurodevelopment outcome between infants with and without growth failure in weight as classified by each growth chart. Additionally, an adjusted model was performed after controlling for SGA that is known to affect neurodevelopment [17]. Unadjusted and adjusted odds ratios with 95% confidence intervals and *p*-values are presented. For each growth reference, the diagnostic performance and discriminatory ability was calculated via area under the receiver operating characteristic curves (AUROC) and compared between the two tools.

## 3. Results

### 3.1. Study Population

**Figure 1:**
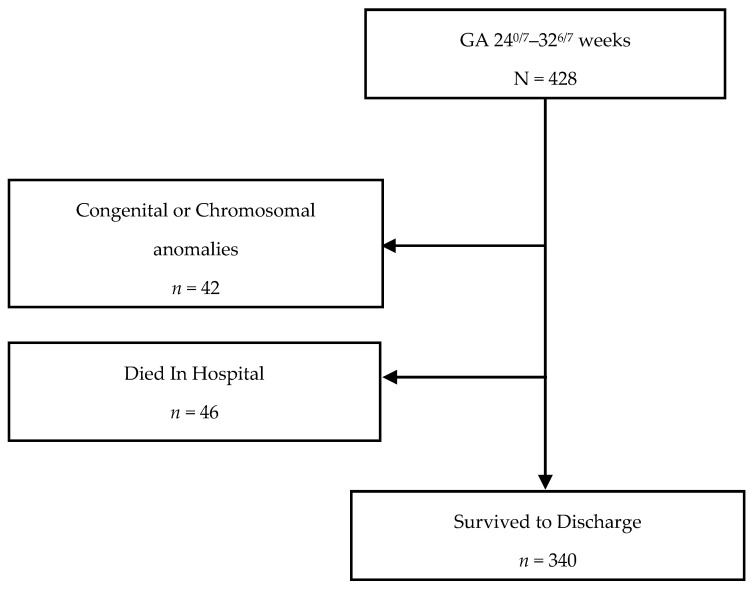
Study population flow chart.

Of the eligible 428 infants born at CHoR between 24^0/7^ and 32^6/7^ weeks of gestation during the study period of January 2014–December 2018, 42 (9.8%) were excluded for congenital or chromosomal abnormalities and 46 (11.9%) died while in hospital. A total of 340 infants who survived to discharge were included and analyzed (Figure 1).

### 3.2. Characteristics of Study Population

The median gestational age at birth was 30 weeks (IQR: 27, 32) with an average birth weight of 1310 g (IQR: 950, 1609) and 55% male infants. Demographics and prevalence of common neonatal morbidities are shown in Table 1.

**Figure 2:**
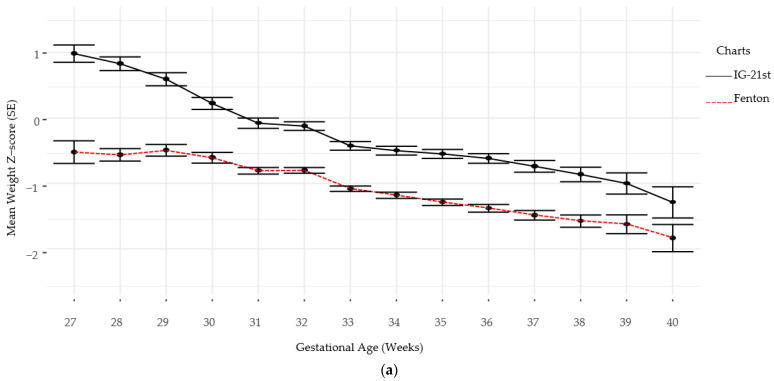

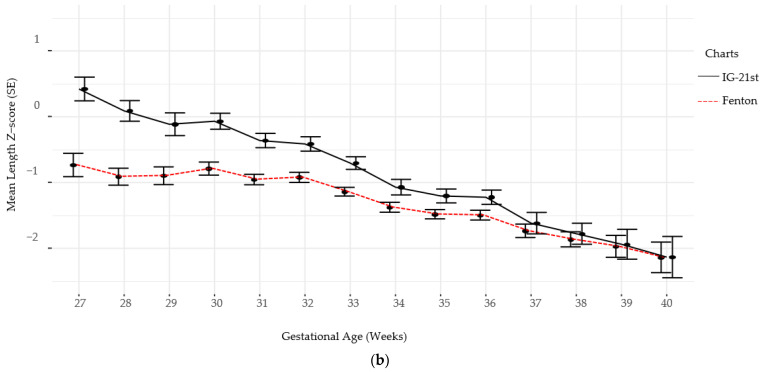
(**a**) Weight Z-scores by gestational age by chart. (**b**) Length Z-scores by gestational age by chart.

Follow-up for standardized neurodevelopmental testing was 37% and 22% at 12 and 24 months, respectively. There was no difference in identifying SGA infants at birth using Fenton versus IG-21st; however, the prevalence of growth failure for both weight and length were higher by Fenton than IG-21st charts (Table 2). The proportion of agreement in classification of SGA was high between the two growth assessment charts, k = 0.94 (0.87, 1.0). However, the agreement in identification of growth failure at discharge was poor for linear growth failure 0.44 (0.35, 0.54) and only moderate for weight growth failure k = 0.60 (0.52, 0.9), (Table 3). Furthermore, there were significant differences in postnatal growth mean z-scores between the two charts at various time points (Figure 2a,b).

The unadjusted logistic regressions (Table 4) between weight-based growth failure for each tool and each of the neurodevelopmental outcomes imply that IG-21st growth failure may be a significant predictor of a poor language development (*p* = 0.094). It is notable that the variability with this association is considerably larger than all other associations of interest as evident by the very wide confidence interval. There appears to be insufficient evidence of any other growth failures (in both tools) being associated with any of the other outcomes of interest. While the AUROC for this near significant predictor is larger than all other predictors (AUROC = 0.608) it is still low implying poor predictive capability of adverse neurodevelopmental outcome in language.

However, as shown by Table 5 below, when adjusting for SGA (by respective tool), the association between weight-based growth failure using IG-21st and adverse language development becomes marginally significant (*p* = 0.049). The odds of a poor neurodevelopmental outcome in language in infants with weight-based growth failure per IG-21st are nearly four times greater (95% CI: 1.01, 26.35) than infants who do not have such growth failure per IG-21st after adjusting for SGA. In terms of predictive capability, it appears that adjusting for SGA slightly improved AUROC (by about 0.05) in both tools for the language neurodevelopmental outcome, while having negligible or no improvement in AUROC for the cognition and motor outcomes. Due to the small event size from small sample size, the variability in the association between the outcomes and SGA in both tools is quite large, leading to very wide confidence intervals. The small event size did not allow for the adjustment of other predictors known to affect neurodevelopmental outcomes such as IVH, BPD, NEC, IVH, and PVL.

## 4. Discussion

Growth charts based on cross-sectional size-at-birth measurements by gestational age should be used cautiously to monitor postnatal growth as intrauterine and extrauterine growth is not comparable owing to the different biological processes, environmental and nutritional approaches that preterm infants are subjected to after birth [18]. In this study, we used two growth standards to evaluate the intrauterine and postnatal growth patterns of preterm infants. The results demonstrated agreement between the two charts in the assessment of size at birth as demonstrated by SGA classification of the study participants. To the contrary, a single center retrospective study by Tuzun et al. has shown that one in four infants classified as SGA by IG-21st were deemed normal by Fenton [19]. In another similar study, prevalence of SGA by IG-21st was higher than by Fenton charts, with short-term neonatal morbidities more strongly associated with those identified by IG-21st [20]. As SGA infants are known to be at increased risk of growth faltering and developing short- and long-term complications, identification of the appropriate growth assessment chart becomes critical in helping clinicians identify infants at risk and institute optimal nutritional plans.

Several definitions have been used to define postnatal growth failure with weight percentiles at discharge below a previously set cut-off value the 10th percentile being the most common one. However, this definition can potentially misclassify infants born SGA who exhibit desirable growth patterns but may be discharged at <10th percentile. Furthermore, other studies have suggested the use of standard deviation scores (Z-score) for longitudinal assessment of growth as it describes growth regardless of centiles at birth and discharge. Based on available literature, we defined growth failure at discharge by a Z-score drop >1 from birth to discharge in weight, linear, or head growth [13,21,22]. In line with previous studies, the results of our study also demonstrated that postnatal growth failure for weight and length were less prevalent with the IG-21st charts as compared to Fenton [19,20,23,24,25,26]. Furthermore, it has been demonstrated that infants with growth impairment by IG-21st were found to have higher short-term morbidities as compared to those defined to have impaired growth by Fenton [23]. In addition to the size at birth and discharge, this study retrospectively compared weekly growth using Fenton and IG-21st and highlights that the difference in growth assessment between the two charts is persistent throughout the hospitalization of the preterm infant. This finding is important as it implies that, with future sufficient evidence to support its’ utility, the appropriate growth-monitoring tool has a potential to be used longitudinally to timely identify growth impairments and inform daily nutritional approaches of preterm infants. While exploring the performance of IG-21st and Fenton charts in classification of preterm infant growth we also examined the long-term functional implication by assessing the ability of the charts to identify the growth impairment predictive of neurodevelopment. The result shows a stronger association between weight-based growth failure as defined by IG-21st and poor language development that was significant when adjusted for SGA status at birth. There was insufficient evidence that weight-based growth failure by either of the chart tools was associated with any of the other neurodevelopmental impairment outcomes. This was validated by the low AUROC implying both charts have poor predictive capability of adverse neurodevelopmental outcomes. This result is contrary to other studies that showed independent associations of growth restriction with adverse neurodevelopment [27,28,29]. We recognize that this finding is likely due to cohort drop out and the small follow-up rate of preterm infants for standard neurodevelopmental assessment which is the main limitation of this study. This excessive missingness may lead to biased results due to making inference from a smaller sample size that may be under-representative of the population of interest (preterm infants in NICU). A strong assumption of missing completely at random (MCAR) was made as opposed to missing not at random (MNAR) since the missing observations were ignored. If the mechanism of missing data was in fact MNAR (e.g., missing evaluations due to poor infant health), then there will be a bias in the results that would underestimate the predictive capabilities of the two tools. Although other neonatal comorbidities including BPD, NEC, IVH, and PVL influence neurodevelopmental outcomes we were unable to adjust for these comorbidities due to study size limitation [30,31,32]. Another limitation is the retrospective study design which did not allow for a complete, more comprehensive, and standardized collection of perinatal, growth, and neurodevelopment data. The study data were also limited to one site which limits the external validity of the study.

Since the development of this growth reference tool, few studies have compared its performance against the fetal-based growth charts commonly used in classifying growth abnormalities, and they fall short of recommending its clinical adoption due to lack of long-term outcome studies on large population samples of preterm infants. This study highlights a potential difference in predictive ability of fetal-based charts versus postnatal growth charts based on healthy preterm longitudinal data. A future study on a large multicenter population-based data to further explore the functional implications of use of specific growth charts in assessing long term outcome risk is warranted.

## 5. Conclusions

In this single center retrospective study, we have demonstrated that postnatal weight and length-based growth failure was less prevalent among preterm infants using IG-21st compared to Fenton charts. The difference in growth is persistent throughout the postnatal period. Weight-based growth failure defined by IG-21st may be more strongly associated with poor neurodevelopmental outcomes than Fenton. Given this significant difference in growth pattern assessment using these two growth charts, we believe future large observational studies are warranted to further delineate the predictive ability of these growth charts in identifying postnatal growth failure that is independently associated with long-term morbidity. This will allow for discerning the relevant functional impact of each tool and support or refute their clinical use.

## Figures and Tables

**Table 1:** Demographic and clinical characteristics of study population.

Characteristics	Median (IQR), Frequency (Percentage) [*n* = 340]
Gestational Age at Birth (weeks)	30 (27, 32)
Birth Weight (g)	1310 (950, 1609)
Birth Length (cm)	39 (35, 41.5)
Head Circumference (cm)	27 (24.2, 28.4)
Male	188 (55.3)
Race	
Black	209 (61.5)
White	98 (28.8)
Other	33 (9.7)
BPD	58 (17.1)
IVH	24 (9.2)
PVL	7 (2.6)
ROP	33 (14.2)
NEC	5 (1.5)
Sepsis	29 (8.6)
Postnatal Steroid	34 (10)
Length of Stay	56.00 (38.8, 90)
Language Neurodevelopmental Deficiency at 12 months [*n* = 122]	68 (55.7)
Cognition Neurodevelopmental Deficiency at 12 months [*n* = 125]	20 (16.0)
Motor Neurodevelopmental Deficiency at 12 months [*n* = 118]	23 (19.5)
Language Neurodevelopmental Deficiency at 24 months [*n* = 75]	38 (50.7)
Cognition Neurodevelopmental Deficiency at 24 months [*n* = 75]	21 (28.0)
Motor Neurodevelopmental Deficiency at 24 months [*n* = 72]	21 (29.2)

BPD—broncho pulmonary dysplasia; IVH—intraventricular hemorrhage; PVL—periventricular leukomalacia. ROP—retinopathy of prematurity; NEC—necrotizing enterocolitis.

**Table 2:** SGA(small for gestational age) and growth failure identification at discharge by chart.

Type *n* = 340	Fenton Frequency (Percentage)	IG-21st Frequency (Percentage)	*p*-Value
SGA ^a^	37 (10.9)	33 (9.7)	0.6137
Growth Failure ^b^			
Weight	127 (39.7)	93 (27.9)	0.0015
Length	215 (67.4)	164 (49.2)	<0.0001
Head Circumference	53 (16.9)	77 (23.5)	0.0395

^a^ SGA defined as birth weight 10th percentile (z score < −1.28); ^b^ growth failure defined as decrease in z-score > 1 from birth to discharge.

**Table 3:** Agreement in prevalence of growth failure by chart.

Growth by Charts	Cohen’s Kappa	95% CI
IG-21st vs. Fenton SGA	0.94	(0.87, 1.00)
IG-21st vs. Fenton Growth Failure (Weight)	0.60	(0.52, 0.69)
IG-21st vs. Fenton Growth Failure (Length)	0.44	(0.35, 0.54)
IG-21st vs. Fenton Growth Failure (Head Cir.)	0.52	(0.40, 0.64)

**Table 4:** Unadjusted analyses of weight-based growth failure as predictor of adverse neurodevelopmental outcome.

	Odds Ratio	95% CI	*p*-Value	AUROC
**Language**				
IG-21st	3.75	(0.96, 24.89)	0.094	0.608
Fenton	1.48	(0.51, 4.67)	0.486	0.547
**Cognition**				
IG-21st	1.35	(0.49, 3.68)	0.557	0.531
Fenton	0.94	(0.34, 2.51)	0.898	0.508
**Motor**				
IG-21st	1.60	(0.57, 4.53)	0.374	0.549
Fenton	1.05	(0.38, 2.89)	0.918	0.506

AUROC: area under the receiver operating characteristic curve.

**Table 5:** SGA(small for gestational age)-adjusted analyses of weight-based growth failure as predictor of adverse neurodevelopmental outcome.

	Odds Ratio	95% CI	*p*-Value	AUROC
**Language**				
Growth Failure IG-21st	3.949	(1.005, 26.351)	0.0490	0.6503
SGA IG-21st	3.962	(0.702, 74.797)	0.1330	0.6503
Growth Failure Fenton	1.545	(0.526, 4.933)	0.4338	0.6073
SGA Fenton	2.939	(0.508, 55.937)	0.2610	0.6073
**Cognition**				
Growth Failure IG-21st	1.442	(0.516, 4.019)	0.4812	0.5887
SGA IG-21st	2.407	(0.741, 8.240)	0.1464	0.5887
Growth Failure Fenton	1.008	(0.363, 0.968)	0.9876	0.5530
SGA Fenton	2.403	(0.644, 9.356)	0.1892	0.5530
**Motor**				
Growth Failure IG-21st	1.618	(0.576, 4.617)	0.3609	0.5480
SGA IG-21st	1.364	(0.381, 4.907)	0.6279	0.5480
Growth Failure Fenton	1.055	(0.379, 2.906)	0.9178	0.5000
SGA Fenton	1.007	(0.233, 3.972)	0.9918	0.5000

**AUROC:** area under the receiver operating characteristic curve.
